# Trajectory of Venezuelan migrant women during prenatal care and childbirth in a city in northern Brazil: a quantitative and qualitative study

**DOI:** 10.1590/0102-311XEN076025

**Published:** 2026-01-09

**Authors:** Leidy Janeth Erazo-Chavez, Thaiza Dutra Gomes de Carvalho, Maria do Carmo Leal, Ana Paula Mesquita Schutz, Laura Froes Nunes da Silva, Rita Suely Bacuri de Queiroz, Zeni Carvalho Lamy

**Affiliations:** 1 Programa de Pós-graduação em Saúde Coletiva, Universidade Federal do Maranhão, São Luís, Brasil.; 2 Universidade Ceuma, São Luís, Maranhão, Brasil.; 3 Escola Nacional de Saúde Pública Sergio Arouca, Fundação Oswaldo Cruz, Rio de Janeiro, Brasil.; 4 Faculdade de Medicina, Universidade Federal do Maranhão, São Luís, Brasil.; 5 Instituto Leônidas e Maria Deane, Fundação Oswaldo Cruz, Manaus, Brasil.

**Keywords:** International Migration, Delivery of Health Care, Prenatal Care, Parturition, Evaluation Study, Migración Internacional, Atención a la Salud, Atención Prenatal, Parto, Estudio de Evaluación

## Abstract

Brazil is the third country that most receives Venezuelan migrants, with approximately 626,000 people. This migratory flow has increased demand on the Brazilian Unified National Health System, especially for care related to pregnancy and childbirth. The aim of the present study was to investigate access to healthcare on the part of Venezuelan migrant women and their perceptions of the care received in Brazil during their prenatal and childbirth care journey. A quantitative-qualitative study was conducted between 2021 and 2023 involving Venezuelan women who reside in the city of Manaus, Amazonas State. In the quantitative component, participant-driven sampling was used (n = 118), with calculation of absolute frequencies and prevalence rates with 95% confidence intervals. The qualitative component involved intentional sampling (n = 39) and Thematic Analysis. In the quantitative results, most participants received prenatal care (95%) mainly at public healthcare services beginning in the first trimester (83%) and with an adequate number of appointments (77%). The majority of births (58%) were natural and 95% of the women had an accompanier. Maternal and neonatal complications were reported in 14% and 21% of cases, respectively. The qualitative component identified difficulties in obtaining exams through the healthcare system and in forming a bond with the maternity ward during prenatal care, travelling from maternity to maternity, and cultural differences that influenced the perceptions of the care received. Language was also a factor that impacted the quality of care. In conclusion, care during pregnancy and childbirth was ensured in the Brazilian universal healthcare system, although challenges persist that require improvement based on the experiences of the migrant women.

## Abstract (es)

Brasil es el tercer país que más recibe migrantes venezolanos, cerca de 626 mil personas. Este flujo migratorio aumentó la demanda en el Sistema Único de Salud, especialmente en la asistencia a la gestación y el parto. Este estudio tuvo como objetivo evaluar el acceso y la percepción de las mujeres migrantes venezolanas sobre la prestación de cuidados de salud recibida en Brasil en su trayectoria asistencial durante el prenatal y el parto. Se trata de un estudio de abordaje cuantitativo y cualitativo, realizado entre 2021 y 2023, con venezolanas residentes en Manaus, Amazonas. En el componente cuantitativo, se utilizó el muestreo dirigido por participante (n = 118), con cálculo de frecuencias absolutas y prevalencias con intervalos de 95% de confianza. En el cualitativo, se adoptó el muestreo intencional (n = 39) y los datos fueron analizados por medio de Análisis Temático. En los resultados cuantitativos, la mayoría de las entrevistadas realizó prenatal (95%) principalmente en unidades públicas, iniciando en el primer trimestre (83%) y con número adecuado de consultas (77%). De los partos, el 58% fueron vaginales y el 95% tuvieron acompañante. Se relataron el 14% de complicaciones maternas y el 21% neonatales. En el componente cualitativo, se identificaron dificultades para realizar exámenes por el sistema de salud y para la vinculación a la maternidad durante el prenatal, la peregrinación preparto entre servicios sanitarios y diferencias culturales que influenciaron las percepciones sobre la asistencia recibida. El idioma también fue un factor que impactó en la calidad de la atención. Se concluye que la asistencia a la gestación y el parto fue garantizada por el sistema de salud brasileño, aunque persisten desafíos que exigen mejoras a partir de las experiencias de las mujeres migrantes.

## Introduction

The intensification of Venezuelan migratory flow to Brazil in recent years has posed a challenge for the Brazilian Unified National Health System (SUS, acronym in Portuguese), particularly in the North Region of the country, where the concentration of this population is greater. Brazil is the third country that receives the most Venezuelan migrants, having received more than 626,000 by 2024, nearly 50% of whom are women ^1^. According to data from the Brazilian National Migration Registration System (SISMIGRA, acronym in Portuguese), approximately 41,000 Venezuelan women resided in the state of Amazonas between 2016 and 2023, 96% of whom lived in the city of Manaus and the majority of these were in the reproductive age range ^2^.

Despite these figures, systematized public data on the use of health services by migrants in the state remain scarce, which reveals a gap in the monitoring and formulation of specific policies for this population. Data from the Brazilian Hospital Information System of the SUS (SIH/SUS, acronym in Portuguese) show that Venezuelan women had 166 births in the state of Amazonas in 2021 and 70 in 2022 ^3^. The lack of reliable data compromises the planning and implementation of specific strategies aimed at improving health care for these migrant women, especially in terms of care offered during pregnancy and childbirth.

Studies show that the lack of health care in Venezuela is one of the reasons for migration ^4^ ^,^ ^5^. According to data on Venezuela from the International Monetary Fund (IMF), infant mortality increased from 14.2% in 2008 to 21% in 2019 and maternal mortality increased from 115 to 125 deaths per 100,000 livebirths between 2013 and 2017 ^6^.

The increase in migratory flow has resulted in greater demands on the SUS, particularly in the capital cities of Boa Vista (Roraima State) and Manaus, which have the highest concentrations of Venezuelans ^7^ ^,^ ^8^. According to Human Rights Watch ^9^, the number of births among Venezuelan women in the city of Boa Vista in the first quarter of 2018 was 2.5 times higher compared to the same period the previous year. Moreover, a survey of Venezuelan women showed that 18.8% did not have adequate access to prenatal care in the city of Manaus ^4^.

Therefore, a better understanding is needed of the quality of prenatal and childbirth care offered to these women in the country. Such an understanding is crucial, considering the importance of this care for the prevention of adverse health outcomes for the mother and infant, for which there is a gap in knowledge.

Understanding how the SUS has responded to the growing demand for care among migrant women, especially in the North Region of the country, is essential to ensuring the right to health and strengthening the principles of equity and integrality. The aim of the present study was to investigate access to healthcare on the part of Venezuelan migrant women and their perceptions of the care received in Brazil during their prenatal and childbirth care journey.

## Method

An evaluative cross-sectional study was conducted with quantitative and qualitative approaches, focusing on the perspective of Venezuelan migrant women who use the SUS. Evaluation processes involving patients who use the system are important and can contribute to health management, as such patients are the subjects of social actions in health and have experiences with high informative potential ^10^. The theoretical approach adopted in this study is anchored on the concept of care trajectory, which enabled reconstructing the experiences of patients who sought care and understanding their choices and decisions throughout this journey in healthcare services ^10^ ^,^ ^11^.

This study is part of an international cooperation project between a university of the United Kingdom and three Brazilian research centers (*Redressing Gendered Health Inequalities of Displaced Women and Girls in Contexts of Protracted Crisis in Central and South America* - ReGHID; https://nascernobrasil.ensp.fiocruz.br/?us_portfolio=reghid). Data collection began in Manaus in 2021, with the quantitative and qualitative components conducted separately. Manaus was chosen because it has received more than 5,500 Venezuelans since April 2018 and has a large concentration of migrant women ^12^.

For the quantitative component, respondent-driven sampling (RDS) ^13^ was employed. Six women, called “seeds”, were selected to initiate recruitment networks. Each seed received three invitations to distribute to three other women in their contact network who met the eligibility criteria of the study (Venezuelan woman 15 to 49 years of age and having migrated to Brazil between 2018 and 2021). This process was successively replicated, resulting in a sample of 755 women. Further details on the sampling method can be found in Szwarcwald et al. ^13^.

A subsample of 118 women eligible to respond to the modules on prenatal care and childbirth was used for the present study. Among these women, 45 were pregnant and 75 had given birth in Brazil (two had given birth in Brazil and were also pregnant at the time of the interview). Interviews addressing sociodemographic characteristics, migration as well as prenatal and delivery variables (Supplementary Material; https://cadernos.ensp.fiocruz.br/static//arquivo/suppl-e00098425_1232.pdf) were conducted in person by Venezuelan interviewers trained by the Oswaldo Cruz Foundation (FIOCRUZ, acronym in Portuguese), using the RedCAP software (https://redcapbrasil.com.br/). Questions on prenatal care were answered by both pregnant women and those who had given birth in Brazil in the previous 12 months. The one-year period was chosen to avoid recall bias.

For data analysis, absolute frequencies and relative prevalence rates with 95% confidence intervals were calculated. All analyses considered the complex sampling process using SPSS software, version 23.0 (https://www.ibm.com/). To calculate the adequacy of the number of prenatal appointments, the minimum of six appointments recommended by the Brazilian Ministry of Health for low-risk pregnancies was considered: at least one appointment in the first trimester, two in the second, and three in the last trimester ^14^.

The qualitative sample was intentional and had the following inclusion criteria: adolescents and women 15 to 49 years of age six months pregnant or more and/or with children born in Brazil less than one year earlier. Indigenous women who did not speak Portuguese or Spanish were excluded. Of the initial sample from 2021 (58 participants), only six met the inclusion criteria. Therefore, a second phase of data collection - conducted between January and March 2023 - was necessary to complete the sample, which was based on saturation (halting the inclusion of new participants when the data begin to repeat) ^15^.

**Figure 1:**
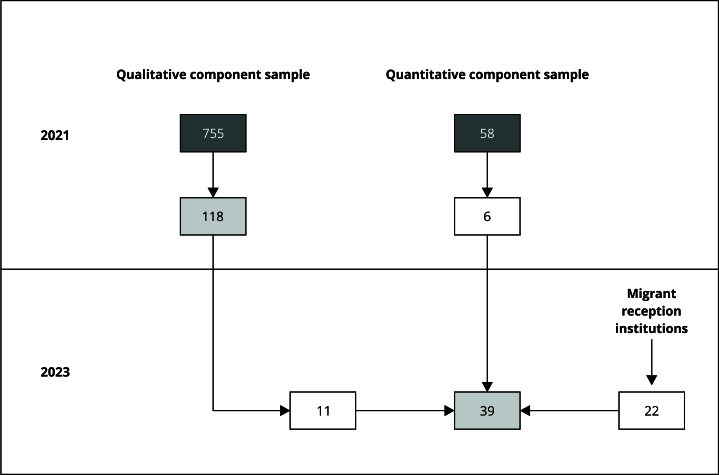
Flowchart of sample of qualitative study. ReGHID, 2021-2023.

Source: prepared by the authors. Nota: representation of flow of sample used in qualitative study developed within ReGHID project between 2021 and 2023. On left, quantitative component began with 755 eligible women, 118 of whom participated in 2021 study (met inclusion criteria). Among these, 11 were contacted for qualitative step in 2023. On right, qualitative component began with 58 participants in 2021, six of whom were included (met inclusion criteria). In 2023, number of participants increased to 39, with 22 linked to migrant reception institutions. Dark gray boxes indicate number of women in sample of umbrella study. White boxes represent participants included in each step. Light gray boxes indicate number of women who met inclusion and exclusion criteria in each step. Arrows represent flow of sample over time and between study components. Migrant reception institutions: nongovernmental organizations and non-profit religious institution - both with social activities in city where study was conducted.

In this second phase, 11 women who had participated in the quantitative interviews and 22 women selected through contact with two migrant reception institutions were interviewed. Thus, the final sample consisted of 39 participants, 13 of whom were pregnant and 26 who had given birth in Brazil. The aim was to encompass diversity in age, ethnicity, schooling, and length of stay in Brazil. Figure 1 displays the flowchart of the participant selection process for the qualitative sample.

Seventeen individual interviews and two focus groups were conducted - one with 10 and the other with 12 participants. A sociodemographic questionnaire, individual interview guide, and focus group guide were used. The interviews were held in settlements, spontaneous land occupations, official migrant shelters, and overnight accommodations for homeless migrants. All interviews were conducted in Spanish, recorded, and subsequently transcribed and analyzed in the original language. The excerpts used in this article were first translated into Portuguese, then English. The data were processed using NVivo software (https://lumivero.com/products/nvivo/). Thematic analysis was performed, which involved the steps of pre-analysis, exploration of the material, and interpretation ^15^.

The study received approval from the Research Ethics Committee of the Federal University of Maranhão (CAAE: 35617020.9.1001.5087). All participants received clarifications regarding the study and signed a statement of informed consent. For those less than 19 years of age, the statement of informed consent was signed by a legal guardian. Fictitious names were used to ensure confidentiality.

## Results

### Description of participants

Most women in both the quantitative and qualitative analyses had self-declared brown skin color, a family income of up to the monthly minimum wage, one to two children, and temporary or permanent residence. Differences were found with regards to some sociodemographic characteristics between the qualitative and quantitative samples, such as age group, marital status, and family income, as shown in Table 1. However, these differences do not imply a statistical comparison between the groups.

**Table 1:** Sociodemographic characteristics and reproductive history of Venezuelan migrant women residing in Manaus, Amazonas, who were pregnant or had a child in Brazil in the previous 12 months. ReGHID, 2021-2023.

Information	Quantitative component (n = 118)			Qualitative component (n = 39)	
	n	%	95%CI	n	%
Sociodemographic					
Age (years)					
15-19	17	14.5	8.6-24.1	6	15.4
20-24	81	68.3	58.3-76.8	9	23.1
25-34	17	14.5	8.5-23.1	21	53.8
35-49	3	2.7	0.8-9.1	3	7.7
Schooling					
≤ Primary school	11	9.2	4.7-17.4	16	41.0
High school	88	74.9	65.1-82.6	16	41.0
≥ Higher education	19	15.9	9.8-24.7	6	15.4
Not informed	0	0.0	-	1	2.6
Race/Skin color					
White	26	21.8	14.6-31.4	10	25.6
Brown	89	76.1	66.4-83.7	22	56.4
Black	1	0.6	0.1-2.8	5	12.8
Indigenous	2	1.4	0.2-9.7	0	0.0
Does not know	0	0.0	-	2	5.1
Marital status in Brazil					
Does not have partner	26	22.0	14.7-31.5	24	61.5
Has partner	92	78.0	68.5-85.3	15	38.5
Family income (minimum wages) *					
No income	3	2.4	0.7-8.0	1	2.6
Up to 1	68	64.5	54.2-73.6	37	94.9
More than 1	35	33.1	24.3-43.4	1	2.6
Type of residence					
Shelter and hostel	11	9.4	4.8-17.6	6	15.4
Rented house	78	66.0	55.7-74.9	33	84.6
Hotel/Lodge	3	2.9	0.8-9.6	0	0.0
Other	26	21.8	14.6-31.2	0	0.0
Migratory status					
Requested refugee status/Refugee	41	35.2	25.2-46.6	13	33.3
Temporary or permanent residence	62	51.8	41.0-62.4	23	59.0
Irregular	15	13.0	7.4-21.9	3	7.7
Number of children					
None	12	10.4	5.3-19.3	6	15.4
1-2	72	60.6	49.7-70.6	24	61.5
3 or more	34	29.0	20.7-39.0	9	23.1
Gestational history					
Migrated pregnant to Brazil					
Yes	27	23.1	15.2-33.5	-	-
No	91	76.9	66.5-84.8	-	-
Became pregnant once after arriving in Brazil					
Yes	92	77.3	67.2-85.0	-	-
No	26	22.7	15.0-32.8	-	-
Pregnant at time of interview					
Yes	45	37.4	27.9-47.9	13	33.3
No	73	62.6	52.1-72.1	26	66.7
Gave birth in Brazil in previous 12 months					
Yes	75	64.9	54.4-74.1	26	66.7
No	43	35.1	25.9-45.6	13	33.3

95%CI: 95% confidence interval.

* 12 chose “does not know/not answer” option.

### Access to prenatal care

Among the women interviewed, 95.8% had at least one prenatal appointment and 83.3% of these women began prenatal care in the first trimester of pregnancy. The main reason for not seeking care was being unaware of the pregnancy (n = 3). Regarding prenatal care, 77.5% had the appropriate number of appointments for gestational age and the majority appointments were at public healthcare services (94.9%). Most of the women women had an ultrasound exam requested during pregnancy and were informed about what service to seek for delivery (79.5%), but only 56.4% of these women created a bond with the maternity hospital by visiting it before delivery (Table 2).

**Table 2:** Information on prenatal care for Venezuelan migrant women residing in Manaus, Amazonas, who were pregnant or had a child in Brazil in the previous 12 months (n = 118). ReGHID, 2021.

Information	n	%	95%CI
Did you have any prenatal appointment			
Yes	113	95.8	89.6-98.4
No	5	4.2	1.6-10.4
Gestational age at beginning of prenatal (n = 113)			
First trimester	95	83.3	73.7-89.9
Second trimester	13	12.0	6.6-20.9
Third trimester	5	4.7	1.8-11.8
Reason for not doing prenatal (for those who did not, n = 5)			
Was unaware of pregnancy	3	45.3	11.4-84.2
Service was distant/difficult access	1	21.1	3.9-63.5
Other reason	2	33.6	5.2-82.4
Number of prenatal appointments adequate for gestational age			
Adequate	87	77.5	67.0-85.4
Inadequate	26	22.5	13.3-31.4
Place of majority of prenatal appointments			
Basic health unit	113	94.9	87.4-98.0
Private clinic	5	5.1	2.0-12.6
Attending professional for majority of appointments *			
Physician	67	62.6	51.5-72.4
Nurse	40	37.4	27.6-48.5
Ultrasound exams requested during prenatal **			
0	1	1.1	0.2-4.9
1	17	15.4	8.6-26.1
2	23	21.0	13.5-31.3
3	31	28.8	20.1-39.5
4	21	19.4	12.2-29.5
5 or more	16	14.1	4.6-17.8
Was informed during prenatal about what service to seek at time of delivery			
Yes	86	79.5	69.3-86.9
No	27	20.5	13.1-30.7
Visited maternity prior to delivery (only for third trimester or those who gave birth in Brazil in previous 12 months, n = 86)			
Yes	51	56.4	43.7-68.4
No	35	43.6	31.6-56.3

95%CI: 95% confidence interval.

* 11 chose “does not know/not answer” option;

** 9 chose does not know/not answer option.

Based on the qualitative interviews, aspects were identified that influenced the perception of prenatal care provided at basic health units (BHU). Difficulty in performing tests, especially ultrasound, was particularly noteworthy. Many expressed a desire to have this test, even in low-risk cases, and opted for the private system, which placed a burden on their already fragile finances.

“*There were some tests that they don’t do in the SUS. So, we also made the sacrifice of doing them separately*” (Eva, 31 years old, white, interview).

“*I paid for most of the ultrasounds myself because they told me it would take two or three months to do them*” (Jurema, 24 years old, brown, interview).

Another aspect mentioned was the lack of guidance on creating a bond with the reference maternity hospital and on general questions during prenatal care.

“*They didn’t explain* [about the bond] *to me. I reached 38 weeks and five days* [of pregnancy]” (Valentina, 27 years old, black, interview).

“*Sometimes I left appointments dissatisfied, because they didn’t answer the questions that I had*” (Jurema, 24 years old, brown, interview).

Despite the negative aspects mentioned by the participants, it was clear that they rated the overall prenatal care that they received in the country positively. In their statements, the women highlighted both the welcoming environment and aspects related to access to tests, appointments, medications, and vitamins as positive aspects of prenatal care in Brazil.

“*My experience here at the prenatal clinic has been excellent. I had health complications, but thank God, the doctors treated me at the right time*” (Camila, 23 years old, brown, focus group).

“*There was no lack of medication. They gave it to me and I had no problems with that*” (Fernanda, 19 years old, white, focus group).

Multiparous women who had experienced prenatal care in Venezuela before the crisis noted differences between the two countries. They felt less welcomed and reported a lack of physical examinations in Brazil. Another common complaint was the current model of alternating appointments between nurses and physicians.

“*In Venezuela, they start measuring your belly beginning with the second month. Yesterday I went for my checkup; it took me longer to get them to see me, and the only thing they did was, ‘Your new prescription so you can get your vitamin’*” (Camila, 23 years old, brown, focus group).

“*If I want an appointment with the doctor, which is what I’m interested in* (...)*, first they refer me to the nurse, who’s really nice, and they run the tests. I have to wait every two months for an appointment with the doctor, whereas it wasn’t like that in Venezuela*” (Carolina, 29 years old, Asian descent, focus group).

### Access to childbirth care

Among those who experienced childbirth in Brazil in the 12 months prior to the survey, 97.8% had singleton pregnancies and delivered in a maternity ward. The majority underwent natural childbirth (57.9%), all resulted in live births, and 14.6% of the infants had a gestational age of less than 37 weeks. Most (95.1%) had an accompanier of their choice during hospitalization for childbirth and the majority of these had an accompanier the entire time (91.4%). More than 14.1% of the migrant women reported some complication during childbirth for the mothers and 21.2% for the newborns (Table 3).

**Table 3:** Delivery information of Venezuelan migrant women residing in Manaus, Amazonas, who gave birth in Brazil in previous 12 months. ReGHID, 2021.

Information	n	%	95%CI
Type of pregnancy			
Singleton	74	97.8	85.5-99.7
Multiple	1	2.2	0.3-14.5
Type of delivery			
Natural	44	57.9	45.1-69.8
Cesarian	31	42.1	30.2-54.9
Delivery outcome			
Live birth	75	100.0	100.0-100.0
Place of delivery *			
Maternity	73	98.9	92.3-99.8
Home	1	1.1	0.2-7.7
Gestational age at delivery (weeks)			
< 37	11	14.6	7.5-26.6
≥ 37	64	85.4	73.4-92.5
Had accompanier for delivery			
Yes	69	95.1	84.6-98.6
No	6	4.9	1.4-15.4
Accompanier remained entire time (n = 69)			
Yes	63	91.4	81.0-96.3
Accompanier did not want to stay	1	0.7	0.1-5.3
Maternity did not allow accompanier to stay	5	7.7	3.0-18.2
Maternal complication during delivery			
Yes	11	14.1	7.5-25.1
No	64	85.9	74.9-92.5
Neonatal complication during delivery			
Yes	16	21.2	12.5-33.8
No	59	78.8	66.2-87.5

95%CI: 95% confidence interval.

* 1 chose “does not know/not answer” option.

Regarding access to hospitals for childbirth, the interviewees statements revealed stories of going from maternity to maternity due to factors such as overcrowding, the difference in the level of complexity of the maternity wards, and the classification of gestational risk.

“*They sent me to Maternity B. I went there when I started urinating blood. There, they told me ‘no’, that if it was an emergency they wouldn’t allow it. There was no room, that I had to go to another maternity*” (Clara, 27 years old, did not declare skin color, focus group).

“*I had to visit all the maternity hospitals in Manaus, I practically* (...) *spent a week in pain*” (Alma, 24 years old, white, interview).

Nonetheless, after entering the maternity wards, most participants spoke highly of the quality of care provided by the healthcare team during labor. Good practices, such as nonpharmacological pain relief, skin-to-skin contact, and breastfeeding within the first hour of life, were offered and well received. Few negative experiences were mentioned, and when they did occur, they were usually related to the attitudes of specific members of the team.

“*The care was exceptional. They helped me with measures so that my pain was less intense*” (Alma, 24 years old, white, interview).

“*When they took the baby out, they put her on my chest*” (Valentina, 27 years old, black, interview).

“*Because of the doctor herself, she treated me unwillingly, but I didn’t pay attention to her*” (Nina, 28 years old, brown, interview).

The participants reported positive experiences during hospitalization, highlighting the availability of medication, food, and newborn care. They also valued newborn screening and exams as important for providing care for the infants.

“*They gave me four antibiotics. A good diet. Everything was clean. My bed was comfortable and there was room for my baby to stay with me. Excellent*” (Blanca, 35 years old, brown, interview).

“*The nurse saw him first and sent him to the pediatrician. She checked his little head, checked to see if he was breathing well, weighed and measured him* (...) *They gave him an ear exam, a vision exam, checked to see if he was pooping normally, examined his tummy, his chest, vaccinated him, and did the heel prick test*” (Carla, 24 years old, brown, interview).

Differences in childbirth care between Venezuela and Brazil were reported by the interviewees. As positive aspects, they highlighted the possibility of delivering without having to pay for supplies and the fact that they could be accompanied, something that they said is not permitted in Venezuela.

“*In Venezuela I had to buy everything, my medication, antibiotics, pain medication, sutures, everything. I bought everything*” (Blanca, 35 years old, brown, interview).

“*In Venezuela, they don’t let you have an accompanier; you’re alone. Not even your mother or anyone else can enter. Here I saw that it was good, that here at least they can enter with you*” (Valentina, 27 years old, black, interview).

On the other hand, some practices employed in Brazil, such as encouraging natural birth and restricting the use of episiotomy and the Kristeller maneuver, were seen as negative by some of the interviewees.

“*I gave birth and they didn’t cut me or anything. I had to push it out myself. My sister, who was with me, helped. I told her ‘get up here so I can be able to do it’, because I know in Venezuela how they help you when you can’t give birth*” (Sofia, 24 years old, brown, focus group).

### Language as a barrier in the care of migrant women

The language barrier affected the women throughout their entire healthcare journey, from access to prenatal to childbirth care, at different times and dimensions of care, impacting their reception and understanding of clinical guidance. Some women expressed fear of continuing prenatal care because they were unable to understand what the healthcare providers were saying. This led, in some cases, to seeking private services with physicians fluent in Spanish, despite the financial hardship.

“*I didn’t go anymore because they speak their language and I don’t understand anything*” (Anita, 26 years old, white, focus group).

“*I haven’t learned much Portuguese yet. So, we made the sacrifice of paying for something private to really understand how everything is with a Colombian* [physician]” (Eva, 31 years old, white, interview).

Furthermore, there were reports of women who felt discriminated against by workers at healthcare services due to the difference in language.

“*I consulted a doctor, who I had check-ups with before I got pregnant and he treated me very badly. He said: ‘You have time here and you have to speak perfect Portuguese, because I lived in Venezuela for a while and I speak perfect Spanish’*” (Jurema, 24 years old, brown, interview).

In contrast, having a healthcare provider fluent in Spanish favored the continuity of care and they felt more confident to ask for further clarifications and guidance.

“*The doctor who was on duty, thank God, spoke three languages: English, Spanish, and Portuguese. He asked me what language. So, I spoke Spanish and he explained the situation to me*” (Carla, 24 years old, brown, interview).

## Discussion

The trajectories of migrant women during prenatal care and childbirth highlight the importance of the SUS in enabling access to health care. The quantitative and qualitative findings of this study complement each other, enabling a deeper understanding of the experiences of Venezuelan women. Both analyses indicate that access to care during the prenatal period and childbirth was ensured. A study previous conducted in the city of Manaus confirms these results, showing that Venezuelan women have free access to universal health care through the SUS, including prenatal care, irrespective of their immigration status ^13^.

This scenario differs from that of other Latin American countries. In Colombia and Peru - which are the main destinations for Venezuelans ^1^ - access to the Colombian General Social Security Health System and the Peruvian Integral Healthcare System, respectively, requires regular immigration status and other administrative requirements. Despite legal exceptions for pregnant women, there is no assurance of the continuity of care or procedures and hospitalization may be charged ^16^. In this study, all women interviewed - even those in an irregular migration situation - received prenatal care and delivery, revealing the importance of global policies that ensure universal access to health care for migrants.

The quantitative component revealed that 23% of the women migrated while pregnant, beginning their journey in Venezuela, where difficulties in gaining access to health care were considerable. Leal et al. ^4^ reported that difficulty in gaining access to health care was the second most cited reason by women regarding their decision to migrate from Venezuela to Brazil.

This study found that prenatal care was primarily provided through the BHU, which were rated as satisfactory due to the welcoming atmosphere and free access to appointments and medications. In the state of Roraima, Venezuelan women also reported positive experiences, demonstrating the importance of universal access to humanized care ^5^. Leal et al. ^4^ found that Venezuelan women used health services almost three times more frequently than Brazilian women. This suggests that the infrastructure mobilized to address migratory flow in the North Region of the country, combined with the existence of the SUS, facilitated access to health care for this population.

Data from the *Birth in Brazil* survey ^17^ conducted with Brazilian women reveal that access to prenatal care through the SUS reaches 99.3% in some places. However, the quality of the care offered remains insufficient, especially in the North Region, where only 10.5% of prenatal appointments were considered adequate. There is a need for improvements in all indicators recommended by the Brazilian Ministry of Health for adequate prenatal care, including early initiation, a minimum number of appointments, essential exams, and establishing a bond with the maternity ward to prevent adverse outcomes for the mother and infant ^17^.

In the case of the Venezuelan migrants who participated in this study, although the overall assessment was positive, the qualitative component revealed dissatisfaction with prenatal care, especially the care model that alternates between nurses and physicians, which was considered odd. The participants also reported a lack of physical examinations, lack of having their questions clarified, and difficulty gaining access to ultrasound exams.

In Brazil, the BHU is the gateway to prenatal care, with alternating appointments between nurses and physicians according to the clinical needs of the women ^14^. This model is in line with guidelines from the Brazilian Ministry of Health ^14^ and World Health Organization (WHO) ^18^, which recognize the strategic role of multidisciplinary teams in providing continuous, humane, cost-effective care during pregnancy and childbirth. However, lack of knowledge on the part of the migrant women with regards to the functioning of the health system in their host country - which is a widely documented fact in the international literature ^19^ - poses an additional challenge for the SUS, potentially generating negative perceptions even when appointments follow national and international protocols.

The quality of prenatal care encompasses various aspects, including physical examinations, effective communication, and the formation of a bond between the healthcare provider and patient. Studies involving Brazilian pregnant women have highlighted weaknesses in these aspects ^20^. In the case of migrant women, overcoming the language barrier is essential to ensuring effective communication ^19^ ^,^ ^21^. Thus, it is all-the-more urgent to ensure comprehensive care that considers the specific needs of migrant women, promotes health education, and strengthens their autonomy.

Most participants in the quantitative study reported having undergone ultrasound, while many in the qualitative study mentioned difficulty accessing the exam. This demonstrates the complementarity of the integrated analysis, which enables understanding the experiences of those who were unable to undergo the exam. Studies involving Venezuelan migrants in the city of Boa Vista also reported difficulties in undergoing this exam ^5^ ^,^ ^22^. The *Birth in Brazil* survey reported less ultrasound coverage in the North Region of the country ^17^. It is important to note, however, that ultrasound only began to be offered in low-risk prenatal care at the end of June 2023, which may explain the reported dissatisfaction with the lack of access ^23^.

Delivery care for the participants in this study was primarily provided at public maternity hospitals, with cesarean section accounting for 42.1%. According to data from the SIH/SUS ^3^, the cesarean section rate in the city of Manaus was 42.8% in 2021, which reflects a practice that exceeds the WHO recommendation (lack of benefit for mother and baby with cesarean section rates above 10% and 15%) ^24^. The high proportion of cesarean sections in the North Region, as observed in Manaus, is a national trend. Pires et al. ^25^ reported a continuous linear increase in cesarean sections in Brazil, which, already ranked second in the world in 2018, occurring in 55.7% of deliveries.

Good practices were mentioned by the participants in the qualitative interviews, including pain management during labor and the offer of food, along with the encouragement of both skin-to-skin contact and breastfeeding. These results are consistent with findings described by Leal et al. ^26^, who identified a significant increase in access to appropriate technologies for labor and birth, especially the use of nonpharmacological methods for pain relief and offering food during labor in the North and Northeast regions.

Another good practice ensured in most cases, as revealed by both the quantitative and qualitative data, was the right to an accompanier during childbirth and hospitalization as part of the benefits of emotional and physical support provided to the woman in labor ^26^. This finding had been demonstrated in another study involving Venezuelan migrants in the state of Roraima ^5^.

However, this right was not always respected. In the quantitative component, five women reported that the maternity hospital denied the presence of an accompanier, which constitutes a serious failure in the recognition of women’s rights. The qualitative data revealed another limitation in the exercise of this right related to the lack of a support network in Brazil. In some cases, the partner could not be present because he was caring for other children. A study of Indian migrants in Australia identified similar experiences, marked by feelings of loneliness, anguish, and guilt due to the separation from emotional support networks ^27^. These findings demonstrate that ensuring social support is crucial to a positive childbirth experience among migrant women.

Some multiparous women reported dissatisfaction with obstetric practices in Brazil - such as the encouragement of natural childbirth and the restriction of episiotomy and the Kristeller maneuver - in the belief that such practices could reduce pain and speed up labor. Makuch et al. ^5^ also noted the surprise of Venezuelan migrants with regards to favoring natural childbirth over cesarean section, which they often expected. These perceptions reveal the influence of previous experiences, individual beliefs, and sociocultural context on the assessment of obstetric care ^28^, underscoring the need for active listening to the experiences and beliefs of pregnant women.

Natural childbirth is valued in Brazil based on evidence that demonstrates reductions in maternal and neonatal morbidity and mortality rates, contributing to a reduction in the occurrence of unnecessary cesarean sections ^17^ ^,^ ^24^ ^,^ ^25^. Episiotomy is indicated in a selective manner, as the routine use of this procedure has no proven benefits and can cause complications ^26^. The Kristeller maneuver is formally contraindicated because it poses risk to both the mother and fetus ^26^. The use of these practices may even constitute obstetric violence ^29^.

The analysis of the data analysis demonstrated that the creation of a bond with the maternity ward, despite guidance during prenatal care, did not materialize for all women surveyed. The quantitative data revealed that four out of every fie women received counseling about the referral maternity ward during prenatal care in primary care, but only half of the women were aware of the service before delivery. The qualitative component expanded this understanding, revealing that in the accounts of going from maternity to maternity, the interviewees had not previously been to the ward, demonstrating that counseling alone is insufficient without effective coordination between the different levels of care.

Another result of the qualitative component that highlights the communication difficulties between levels of care was that few women had received guidance on post-discharge care and none received home visits during the prenatal and/or postpartum periods.

**Figure 2:**
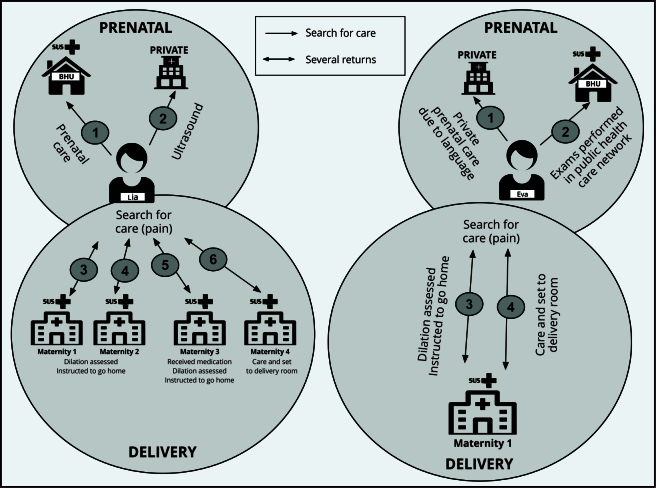
Trajectories of prenatal and childbirth care of migrant Venezuelan women: cases of Lia and Eva. ReGHID, 2021-2023.

The lack of adequate guidance and the refusal of care resulted in experiences of distress for some women, such as Lia, who, despite having received prenatal care through the SUS, had to turn to the private sector for exams. In the last week of pregnancy and during labor, she visited four maternity hospitals without having her needs met. At the first two, she was only assessed for a lack of dilation and instructed to return home. At the third, she received pain medication. She was admitted to the fourth hospital, with dilation already at 8cm, and described a prolonged, difficult labor. The newborn had respiratory complications and required resuscitation (Figure 2).

BHU: basic health unit; SUS: Brazilian Unified National Health Sytem. Source: prepared by the authors, based on Medeiros et al. ^10^. Note: representation of care-seeking trajectories of two Venezuelan migrant women during prenatal care and childbirth in city in northern Brazil. Lia’s case (left) shows multiple searches for care in both public and private sectors, including four different maternity hospitals at time of delivery. Eva’s case (right) reveals search for private prenatal care due to language difficulties and tests through public health system, with two appointments at same maternity hospital at time of delivery. Arrows indicate flow and return to services. Number indicate chronological order of searches for care. Continuous arrows represent flow of care; double arrows indicate multiple returns.

Wandering among maternities in the prepartum period persists in Brazil, especially in the North region of the country, and is associated with worse neonatal outcomes ^30^. Studies indicate that this experience also affects Brazilian women ^17^ ^,^ ^30^, revealing persistent weaknesses in the SUS. Among migrants, wandering may result from a lack of prior connection to a referral maternity hospital, in addition to financial, administrative, and cultural barriers ^19^. Forming a bond with the maternity hospital during prenatal care is a strategy to ensure comprehensive care that helps reduce wandering and provides security for pregnant women ^17^.

The language barrier also exerts an impact on care, as in the case of Eva, who sought private prenatal care but had some tests performed through the SUS, paying for those that were not available. During labor, she received initial care from nurses, but with the shift change, she felt insecure and reported a lack of attention to her pain. With low platelet counts, she was transferred to the emergency room, where she underwent an episiotomy with no clear justification from the physician (Figure 2).

The language barrier hinders the exchange of information with healthcare providers and the understanding of how the healthcare system in host the country works ^19^ ^,^ ^21^. This situation underscores the need to strengthen the SUS in terms of funding, infrastructure, and training to improve prenatal and childbirth care for this population. Moreover, the linguistic discrimination reported by the interviewees has been reported in other studies, with the blaming of patients for not understanding Portuguese ^8^ ^,^ ^21^ ^,^ ^28^.

Discrimination can constitute a significant obstacle to gaining access to healthcare services, depriving migrants of their rights and negatively impacting their lives. Strategies for overcoming language barriers, such as the assistance of professional translators and/or the training and inclusion of migrants as community health agents, can improve the relationship between these patients and healthcare staff, thus improving the care provided and the experience with the healthcare system ^19^ ^,^ ^31^.

These experiences demonstrate how communication difficulties between healthcare providers and pregnant women, combined with a lack of coordination between the different levels of care, compromise comprehensive care. For Venezuelan women, who face cultural and linguistic challenges, these weaknesses in the SUS are even more complex.

### Strengths and limitations of study

The strengths of this study include the combination of quantitative and qualitative approaches, the use of RDS in the quantitative component to gain access to a hard-to-reach population, and the reconstruction of the care trajectory across both components, which enabled an comprehensive view of the care received. The limitations include the exclusion of Indigenous women who did not speak Portuguese or Spanish, which may have limited the understanding of the specific experiences of this group, and the lack of data extracted from the health records of the pregnant women, considering the possibility of recall bias, especially among those who gave birth approximately 12 months earlier.

## Conclusion

This study identified the socioeconomic, cultural, and reproductive characteristics of Venezuelan migrant women in Brazil and identified barriers to accessing healthcare. The analysis focused on the pregnancy-postpartum period highlighted the importance of the SUS in welcoming and promoting the health of the migrant population, thus contributing to public health by identifying their needs and proposing ways to improve care.

The trajectory of these women was marked by both positive and negative aspects. In general, the interviewees expressed satisfaction with the care received during the prenatal period and childbirth, with access to appointments, exams, medications, and good practices, such as the presence of an accompanier, skin-to-skin contact, and the encouragement of breastfeeding. Despite this positive assessment, the participants also reported problems already existing in the SUS, such as difficulties in establishing a bond with the maternity ward during prenatal care, having to wander among maternities before receiving care, and communication failures between the different levels of care. Moreover, the language barrier impacted all phases of care and intensified the challenges.

## Data Availability

The research data are available upon request to the corresponding author.
