# Supplementary material for: Trajectory of Venezuelan migrant women during prenatal care and childbirth in a city in northern Brazil: a quantitative and qualitative study
Source: Cad Saude Publica. 2026 Jan 9;41(12):e00076025. doi: 10.1590/0102-311XEN076025 (PMC12799136; doi:10.1590/0102-311XEN076025)
Supplement: Supplementary Material [file 1678-4464-csp-41-12-EN076025-s.pdf]

MATERIAL SUPLEMENTAR

Variaveís incluidas na entrevista estruturada do componente quantitativo. ReGHID, 2021.

Variaveís Entrevista Estruturada
**Variáveis sociodemográficas e migratórias:** Idade (categorias :15 a 19 anos; 20 a 24 anos; 25 a 34 anos e 35 a 49 anos); Escolaridade (categorias: até o Ensino fundamental, Ensino médio e Ensino superior completo ou mais); Raça/cor (categorias: Branca, Parda, Preta, Indígena); Situação conjugal (Não tem companheiro(a)/Tem companheiro(a)); Renda familiar (categorias: Sem renda, Até 1 salário mínimo (SM), acima de 1 SM); Tipo de moradia (categorias: abrigo/albergue, Casa Alugada, Hotel/Hostel, Outro); Status migratório (categorias: Solicitante de refúgio/Refugiada, Residente temporário ou permanente, Irregular); Número de filhos (categorias: Nenhum filho, 1 a 2 filhos e 3 ou mais filhos); Migrou gestante para o Brasil (categorias: Sim e Não); Ficou grávida alguma vez depois de chegar ao Brasil (categorias: Sim e Não); Status gestacional no momento da entrevista (categorias: Sim e Não); Tiveram filho no Brasil nos últimos 12 meses (categorias: Sim e Não).
**Variáveis sobre o pré-natal:** Fez alguma consulta de PN (Sim, Não); Idade Gestacional de início de PN (Primeiro trimestre, Segundo trimestre, Terceiro Trimestre); Motivo para não fazer PN (somente para as que relataram que não fizeram nenhuma consulta de PN) (categorias: Não sabia que estava grávida, Serviço era distante/difícil acesso, Outro motivo); Número de consultas de pré-natal adequado para idade gestacional (Adequado, Não adequado); Local onde realizou a maioria das consultas de PN (categorias: Unidade Básica de Saúde e Clínica particular); Profissional que atendeu na maioria das consultas (categorias: Médico e Enfermeiro); Número de exames de ultrassonografia solicitados pelo PN (categorias: zero, 1, 2, 3, 4, 5 ou mais); Durante o PN foi informada sobre qual serviço buscar na hora do parto (categorias: Sim ou Não); Visitou a maternidade antes do parto (só para terceiro trimestre ou que tiveram filho no Brasil nos últimos 12 meses) (Categorias: Sim e Não).
**Variáveis sobre o parto:** Tipo de gestação (categorias: Única e Gemelar); Tipo de parto (categorias: Vaginal e Cesariana); Desfecho do parto (categorias: Nascido vivo, Natimorto); Local do parto (categorias: Maternidade e Casa); Idade gestacional do parto (categorias: menos de 37 semanas e igual ou maior que 37 semanas); Teve acompanhante para o parto (Sim ou Não); Acompanhante ficou durante todo o período (categorias: acompanhante ficou durante todo o tempo (categorias: Sim, Não tinha acompanhante, O acompanhante não quis ficar, A maternidade não deixou ficar); A mãe teve alguma complicação no parto (categorias: Sim e Não); O recém-nascido teve alguma complicação no parto (categorias: Sim e Não).
