# Supplementary material for: Trajectory of Venezuelan migrant women during prenatal care and childbirth in a city in northern Brazil: a quantitative and qualitative study
Source: Cad Saude Publica. 2026 Jan 9;41(12):e00076025. doi: 10.1590/0102-311XEN076025 (PMC12799136; doi:10.1590/0102-311XEN076025)
Supplement: Translation

# Translation: Trajetória assistencial de mulheres migrantes venezuelanas durante o pré-natal e o parto em uma cidade do norte do Brasil: um estudo de abordagem quantitativa e qualitativa

**DOI:** 10.1590/0102-311XPT076025

**Authors:** Leidy Janeth Erazo-Chavez, Thaiza Dutra Gomes de Carvalho, Maria do Carmo Leal, Ana Paula Mesquita Schutz, Laura Froes Nunes da Silva, Rita Suely Bacuri de Queiroz, Zeni Carvalho Lamy

**Affiliations:** 1 Programa de Pós-graduação em Saúde Coletiva, Universidade Federal do Maranhão, São Luís, Brasil.; 2 Universidade Ceuma, São Luís, Maranhão, Brasil.; 3 Escola Nacional de Saúde Pública Sergio Arouca, Fundação Oswaldo Cruz, Rio de Janeiro, Brasil.; 4 Faculdade de Medicina, Universidade Federal do Maranhão, São Luís, Brasil.; 5 Instituto Leônidas e Maria Deane, Fundação Oswaldo Cruz, Manaus, Brasil.

**Keywords:** Migração Internacional, Atenção à Saúde, Cuidado Pré-Natal, Parto, Estudo de Avaliação

## Abstract

O Brasil é o terceiro país que mais recebe migrantes venezuelanos, com cerca de 626 mil pessoas. Esse fluxo migratório aumentou a demanda no Sistema Único de Saúde, especialmente na assistência à gestação e ao parto. Este estudo objetivou avaliar o acesso e a percepção das mulheres migrantes venezuelanas sobre a prestação de cuidados de saúde recebida no Brasil, em sua trajetória assistencial durante o pré-natal e o parto. Trata-se de uma pesquisa de abordagem quantitativa e qualitativa, realizada entre 2021 e 2023, com venezuelanas residentes em Manaus, Amazonas. No componente quantitativo, utilizou-se a amostragem dirigida por participante (n = 118), com cálculo de frequências absolutas e prevalências com intervalos de 95% de confiança. No qualitativo, adotou-se a amostragem intencional (n = 39), e os dados foram analisados por meio de Análise Temática. Nos resultados quantitativos, a maioria das entrevistadas realizou pré-natal (95%), principalmente em unidades públicas, iniciando-o no primeiro trimestre (83%) e com número adequado de consultas (77%). Dos partos, 58% foram vaginais e 95% contaram com acompanhante. Foram relatadas 14% de complicações maternas e 21% neonatais. No componente qualitativo, identificaram-se dificuldades para a realização de exames pelo sistema de saúde e para a vinculação à maternidade durante o pré-natal, além de peregrinação anteparto e diferenças culturais que influenciaram as percepções sobre a assistência recebida. O idioma também foi um fator que impactou a qualidade do atendimento. Conclui-se que a assistência à gestação e ao parto foi garantida pelo sistema de saúde brasileiro, embora persistam desafios que exigem aprimoramento a partir das experiências das mulheres migrantes.

## Introdução

A intensificação do fluxo migratório venezuelano para o Brasil nos últimos anos tem desafiado o Sistema Único de Saúde (SUS), particularmente na Região Norte, onde há maior concentração dessa população. O Brasil é o terceiro país que mais recebe migrantes venezuelanos, tendo recebido mais de 626 mil até 2024, e destes, quase 50% são mulheres ^1^. De acordo com dados extraídos do Sistema de Registro Nacional Migratório (SISMIGRA), aproximadamente 41 mil mulheres venezuelanas residiam no Estado do Amazonas entre 2016 e 2023, das quais 96% na cidade de Manaus, sendo a maioria em idade reprodutiva ^2^.

Apesar desses números, ainda são escassos os dados públicos e sistematizados sobre a utilização de serviços de saúde por migrantes no estado, o que evidencia uma lacuna no monitoramento e na formulação de políticas específicas para essa população. Dados do Sistema de Informações Hospitalares do SUS (SIH/SUS) demonstram que ocorreram 166 partos de mulheres venezuelanas em 2021 e 70 em 2022 no Estado do Amazonas ^3^. A ausência de dados fidedignos compromete o planejamento e a implementação de estratégias específicas voltadas à melhoria da assistência à saúde dessas mulheres migrantes, em especial no que tange ao cuidado durante a gestação e o parto. 

Estudos mostram que um dos motivos para a migração foi a falta de assistência à saúde na Venezuela ^4^ ^,^ ^5^. Segundo dados do Fundo Monetário Internacional (FMI) sobre a Venezuela, a mortalidade infantil aumentou de 14,2% em 2008 para 21% em 2019, e a mortalidade materna de 115 para 125 mortes por 100 mil nascidos vivos entre 2013 e 2017 ^6^.

O aumento do fluxo migratório resultou em maior demanda do SUS, em particular nas capitais Boa Vista (Roraima) e Manaus, que concentram o maior número de venezuelanos ^7^ ^,^ ^8^. Segundo o Human Rights Watch ^9^, no primeiro trimestre de 2018, o número de partos de mulheres venezuelanas foi 2,5 vezes maior que no mesmo período do ano anterior na cidade de Boa Vista. Uma pesquisa realizada com mulheres venezuelanas mostrou que 18,8% não tiveram acesso adequado ao pré-natal na cidade de Manaus ^4^.

Assim, é necessário compreender melhor a qualidade da assistência ao pré-natal e ao parto oferecida a essas mulheres no país. Tal compreensão é fundamental, considerando a relevância desse cuidado para a prevenção de desfechos adversos à saúde da díade mãe-bebê, que ainda representam uma lacuna do conhecimento.

Nesse cenário, compreender como o SUS tem respondido à crescente demanda por assistência das mulheres migrantes no sistema de saúde brasileiro, sobretudo na Região Norte, é essencial para assegurar o direito à saúde e fortalecer os princípios de equidade e integralidade do sistema. Este estudo avaliou o acesso e a percepção das mulheres migrantes venezuelanas sobre a prestação de cuidados de saúde recebida no Brasil em sua trajetória assistencial durante o pré-natal e o parto.

## Método

Trata-se de uma pesquisa avaliativa transversal, de abordagem quantitativa e qualitativa, centrada na perspectiva de mulheres migrantes venezuelanas usuárias do SUS. Processos avaliativos que envolvem usuárias são importantes e podem contribuir para a gestão em saúde, considerando que são sujeitos da ação social em saúde e portadoras de experiências com alta potência formativa ^10^. Nessa perspectiva, a abordagem teórica adotada neste estudo está ancorada no conceito de trajetória assistencial, que permitiu reconstruir a vivência das usuárias na busca por cuidado, compreendendo suas escolhas e decisões ao longo desse percurso nos serviços de saúde ^10^ ^,^ ^11^.

Este estudo faz parte de um projeto de cooperação internacional entre uma universidade do Reino Unido e três centros de pesquisa do Brasil (*Redressing Gendered Health Inequalities of Displaced Women and Girls in Contexts of Protracted Crisis in Central and South America* - ReGHID; https://nascernobrasil.ensp.fiocruz.br/?us_portfolio=reghid), cuja coleta de dados foi iniciada em Manaus, em 2021, com os componentes quantitativo e qualitativo conduzidos de forma independente. Manaus foi escolhida por ter recebido, desde abril de 2018, mais de 5.500 venezuelanos, com grande concentração de mulheres migrantes ^12^.

Para o componente quantitativo, utilizou-se o método de Amostragem Dirigida pelo Participante, ou em inglês, *Respondent Driven Sampling* (RDS) ^13^. Seis mulheres, chamadas de “sementes”, foram selecionadas para iniciar as redes de recrutamento. Cada “semente” recebeu três convites para distribuir a outras três mulheres de sua rede de contatos que atendiam aos critérios de elegibilidade do estudo (ser mulher venezuelana, ter de 15 a 49 anos de idade e ter migrado para o Brasil entre 2018 e 2021). Esse processo foi sucessivamente replicado, resultando em uma amostra de 755 mulheres. Detalhes adicionais sobre o método de amostragem podem ser encontrados em Szwarcwald et al. ^13^.

Para este trabalho, utilizou-se uma subamostra de 118 mulheres que eram elegíveis para responder os módulos sobre pré-natal e parto, das quais 45 eram gestantes e 75 haviam tido filhos no Brasil (duas haviam tido filhos no Brasil e estavam grávidas no momento da entrevista). Entrevistas estruturadas, com variáveis sociodemográficas, migratórias e sobre o pré-natal e parto (Material Suplementar; https://cadernos.ensp.fiocruz.br/static//arquivo/suppl-e00098425_1232.pdf), foram aplicadas presencialmente por entrevistadoras venezuelanas treinadas pela Fundação Oswaldo Cruz (FIOCRUZ), realizadas com uso do software REDCap (https://redcapbrasil.com.br/). As perguntas sobre pré-natal foram respondidas tanto pelas gestantes quanto por aquelas que tiveram filhos no Brasil nos últimos 12 meses. O período de um ano foi determinado a fim de evitar viés de memória.

Para a análise dos dados, foram calculadas frequências absolutas e prevalências relativas com intervalos de 95% de confiança. Todas as análises consideraram o processo complexo de amostragem, utilizando-se o software SPSS, versão 23.0 (https://www.ibm.com/). Para o cálculo da variável de adequação do número de consultas de pré-natal, considerou-se o calendário mínimo de seis consultas preconizado pelo Ministério da Saúde do Brasil para gestação de risco habitual, ou seja, a realização de pelo menos uma consulta no primeiro trimestre, duas no segundo e três no último trimestre de gestação ^14^.

A amostra qualitativa foi intencional, tendo como critérios de inclusão: mulheres de 15 a 49 anos, com seis meses ou mais de gestação e/ou com filhos nascidos no Brasil há menos de um ano. Foram excluídas aquelas de etnia indígena que não falavam português ou espanhol. Da amostra inicial de 2021 (58 participantes), apenas seis cumpriram os critérios de inclusão. Dessa forma, uma segunda fase de coleta de dados, realizada entre janeiro e março de 2023, foi necessária para o fechamento amostral por saturação, que interrompe a inclusão de novos participantes quando os dados começam a se repetir ^15^.

Nesta segunda fase, foram entrevistadas 11 mulheres que haviam participado das entrevistas do quantitativo e 22 mulheres selecionadas a partir do contato com duas instituições de acolhimento aos migrantes. Assim, a amostra final foi de 39 participantes, sendo 13 gestantes e 26 que haviam tido filhos no Brasil. Buscou-se abranger diversidade em idade, etnia, escolaridade e tempo de permanência no Brasil. A Figura 1 apresenta o fluxograma da seleção dos participantes da amostra qualitativa.

**Figura 1:** Fluxograma de amostra do estudo qualitativo. ReGHID, 2021-2023.

Fonte: elaboração própria. Nota: representação do fluxo da amostragem utilizada no estudo qualitativo desenvolvido no âmbito do projeto ReGHID entre 2021 e 2023. À esquerda, o componente quantitativo teve início com 755 mulheres elegíveis, das quais 118 participaram da pesquisa em 2021 (por cumprirem os critérios de inclusão e exclusão). Dessas, 11 foram contatadas para a etapa qualitativa em 2023. À direita, o componente qualitativo iniciou com 58 participantes em 2021, das quais 6 foram incluídas (por cumprirem os critérios de inclusão e exclusão). Em 2023, o número de participantes aumentou para 39, sendo 22 vinculadas a instituições de acolhimento a migrantes. As caixas cinza-escuro indicam o número de mulheres da amostra do estudo guarda-chuva. As caixas brancas representam participantes incluídas em cada etapa. As caixas em cinza-claro indicam o número de mulheres elegíveis que atenderam aos critérios de inclusão e exclusão em cada etapa. As setas representam o fluxo da amostragem ao longo do tempo e entre componentes do estudo. Instituições de acolhimento aos migrantes: uma organização não-governamental e uma instituição religiosa sem fins lucrativos, ambas com atuação social na cidade do estudo.

As técnicas utilizadas foram entrevistas individuais e em grupo. Foram realizadas 17 entrevistas individuais do tipo semiestruturadas e dois grupos focais, sendo um com 10 e outro com 12 participantes. Os instrumentos utilizados foram questionário sociodemográfico, roteiro de entrevistas individuais semiestruturadas e roteiro de grupos focais. As entrevistas ocorreram em assentamentos, ocupações espontâneas, abrigos oficiais para migrantes e acomodações de pernoite para migrantes em situação de rua. Todas as entrevistas foram realizadas em espanhol, gravadas e posteriormente transcritas e analisadas no idioma original. Os trechos utilizados neste artigo foram traduzidos para o português.

Os dados coletados foram processados com o apoio do software NVivo (https://lumivero.com/products/nvivo/). Foi realizada Análise Temática que abrangeu as seguintes etapas: pré-análise, exploração do material e interpretação ^15^.

O estudo foi aprovado no Comitê de Ética em Pesquisa da Universidade Federal do Maranhão (CAAE; 35617020.9.1001.5087). Todos os participantes receberam informações sobre a pesquisa e assinaram o Termo de Consentimento Livre e Esclarecido (TCLE). Para menores de 19 anos, o responsável legal assinou Termo de Assentimento Livre e Esclarecido (TALE). Para garantir o sigilo, foram utilizados nomes fictícios.

## Resultados

### Descrição das participantes

A maioria das mulheres, tanto do componente quantitativo quanto do qualitativo, se autodeclarou parda, com renda familiar mensal de até 1 salário mínimo, 1 a 2 filhos e residência temporária ou permanente. De forma descritiva, foi possível observar que algumas outras características sociodemográficas das etapas qualitativa e quantitativa - como faixa etária, situação conjugal e renda familiar - se diferenciaram, como pode ser visto na Tabela 1. Ressalta-se que essas diferenças não implicam em comparação estatística entre os grupos.

**Tabela 1:** Informações sociodemográficas e histórico reprodutivo de migrantes venezuelanas residentes em Manaus, Amazonas, que estavam gestantes ou que tiveram filho no Brasil nos últimos 12 meses. ReGHID, 2021-2023.

Informações	Componente quantitativo (n = 118)			Componente qualitativo (n = 39)	
	n	%	IC95%	n	%
Sociodemográficas					
Idade (anos)					
15-19	17	14,5	8,6-24,1	6	15,4
20-24	81	68,3	58,3-76,8	9	23,1
25-34	17	14,5	8,5-23,1	21	53,8
35-49	3	2,7	0,8-9,1	3	7,7
Escolaridade					
≤ Ensino Fundamental	11	9,2	4,7-17,4	16	41,0
Ensino Médio	88	74,9	65,1-82,6	16	41,0
≥ Ensino Superior	19	15,9	9,8-24,7	6	15,4
Não informado	0	0,0	-	1	2,6
Raça/Cor					
Branca	26	21,8	14,6-31,4	10	25,6
Parda	89	76,1	66,4-83,7	22	56,4
Preta	1	0,6	0,1-2,8	5	12,8
Indígena	2	1,4	0,2-9,7	0	0,0
Não sabe	0	0,0	-	2	5,1
Situação conjugal no Brasil					
Não tem companheiro(a)	26	22,0	14,7-31,5	24	61,5
Tem companheiro(a)	92	78,0	68,5-85,3	15	38,5
Renda familiar (salários mínimos) *					
Sem renda	3	2,4	0,7-8,0	1	2,6
Até 1	68	64,5	54,2-73,6	37	94,9
Acima de 1	35	33,1	24,3-43,4	1	2,6
Tipo de moradia					
Abrigo e albergue	11	9,4	4,8-17,6	6	15,4
Casa alugada	78	66,0	55,7-74,9	33	84,6
Hotel/*Hostel*	3	2,9	0,8-9,6	0	0,0
Outro	26	21,8	14,6-31,2	0	0,0
*Status* migratório					
Solicitante de refúgio/Refugiada	41	35,2	25,2-46,6	13	33,3
Residência temporária ou permanente	62	51,8	41,0-62,4	23	59,0
Irregular	15	13,0	7,4-21,9	3	7,7
Número de filhos					
Nenhum	12	10,4	5,3-19,3	6	15,4
1-2	72	60,6	49,7-70,6	24	61,5
3 ou mais	34	29,0	20,7-39,0	9	23,1
Histórico gestacional					
Migrou gestante para o Brasil					
Sim	27	23,1	15,2-33,5	-	-
Não	91	76,9	66,5-84,8	-	-
Ficou grávida alguma vez depois de chegar ao Brasil					
Sim	92	77,3	67,2-85,0	-	-
Não	26	22,7	15,0-32,8	-	-
*Status* gestacional no momento da entrevista					
Sim	45	37,4	27,9-47,9	13	33,3
Não	73	62,6	52,1-72,1	26	66,7
Tiveram filho no Brasil nos últimos 12 meses					
Sim	75	64,9	54,4-74,1	26	66,7
Não	43	35,1	25,9-45,6	13	33,3

IC95%: intervalo de 95% de confiança.

* 12 responderam a opção não sabe/não responde.

### Acesso e assistência no pré-natal

Entre as mulheres migrantes entrevistadas, 95,8% realizaram alguma consulta de pré-natal e, dentre essas, 83,3% iniciaram o pré-natal no primeiro trimestre de gestação. O principal motivo para não ter buscado assistência foi não saber que estava grávida (n = 3). Ainda sobre o pré-natal, 77,5% realizaram o número de consultas adequado para idade gestacional, sendo a maioria em unidades públicas de saúde (94,9%). A maior parte das mulheres teve exame de ultrassonografia solicitado durante a gestação e foi informada sobre qual serviço buscar para o atendimento ao parto (79,5%), mas apenas 56,4% dessas estabeleceram vínculo com a maternidade, realizando visita antes do parto (Tabela 2).

**Tabela 2:** Informações sobre o pré-natal de mulheres migrantes venezuelanas residentes em Manaus, Amazonas, que estavam gestantes ou que tiveram filho no Brasil nos últimos 12 meses (n = 118). ReGHID, 2021.

Informações	n	%	IC95%
Fez alguma consulta de pré-natal			
Sim	113	95,8	89,6-98,4
Não	5	4,2	1,6-10,4
Idade gestacional de início de pré-natal (n = 113)			
Primeiro trimestre	95	83,3	73,7-89,9
Segundo trimestre	13	12,0	6,6-20,9
Terceiro trimestre	5	4,7	1,8-11,8
Motivo para não fazer pré-natal (para as que não fizeram pré-natal, n = 5)			
Não sabia que estava grávida	3	45,3	11,4-84,2
Serviço era distante/difícil acesso	1	21,1	3,9-63,5
Outro motivo	2	33,6	5,2-82,4
Número de consultas de pré-natal adequado para idade gestacional			
Adequado	87	77,5	67,0-85,4
Inadequado	26	22,5	13,3-31,4
Local onde realizou a maioria das consultas de pré-natal			
Unidade básica de saúde	113	94,9	87,4-98,0
Clínica particular	5	5,1	2,0-12,6
Profissional que atendeu na maioria das consultas *			
Médico	67	62,6	51,5-72,4
Enfermeiro	40	37,4	27,6-48,5
Exames de ultrassonografia solicitados durante o pré-natal **			
0	1	1,1	0,2-4,9
1	17	15,4	8,6-26,1
2	23	21,0	13,5-31,3
3	31	28,8	20,1-39,5
4	21	19,4	12,2-29,5
5 ou mais	16	14,1	4,6-17,8
Durante o pré-natal, foi informada sobre qual serviço buscar na hora do parto			
Sim	86	79,5	69,3-86,9
Não	27	20,5	13,1-30,7
Visitou a maternidade antes do parto (só para terceiro trimestre ou que tiveram filho no Brasil nos últimos 12 meses, n = 86)			
Sim	51	56,4	43,7-68,4
Não	35	43,6	31,6-56,3

IC95%: intervalo de 95% de confiança.

* 11 responderam a opção não sabe/não responde;

** 9 responderam a opção não sabe/não responde.

A partir dos relatos das entrevistas qualitativas, foram identificados aspectos que interferiram na percepção do cuidado oferecido durante o pré-natal nas unidades básicas de saúde (UBS). Destaca-se a dificuldade na realização de exames, principalmente a ultrassonografia. Muitas mulheres manifestaram o desejo de realizar esse exame, mesmo em casos de risco habitual, e optaram pelo sistema privado, o que gerou ônus para suas economias já fragilizadas.

“*Houve alguns exames que eles não fazem no SUS. E aí também fizemos o sacrifício de fazer à parte*” (Eva, 31 anos, branca, entrevista).

“*Fiz a maior parte dos ultrassons por conta própria, porque me disseram que demoraria dois ou três meses para ser feito*” (Jurema, 24 anos, parda, entrevista).

Outro aspecto mencionado foi a falta de orientações sobre a vinculação à maternidade de referência e sobre dúvidas gerais durante o pré-natal.

“*Eles não me explicaram* [sobre a vinculação]*. Cheguei a 38 semanas e 5 dias* [de gestação]” (Valentina, 27 anos, negra, entrevista).

“*Às vezes eu saía das consultas insatisfeita, porque não esclareciam as dúvidas que eu tinha*” (Jurema, 24 anos, parda, entrevista).

Apesar dos pontos negativos referidos pelas entrevistadas, foi possível compreender que, em geral, elas avaliaram positivamente o atendimento ao pré-natal recebido no país. As mulheres destacaram, em suas, falas tanto questões de acolhimento quanto aspectos relacionados ao acesso a exames, consultas, medicações e vitaminas como pontos positivos no atendimento pré-natal no Brasil.

“*Minha experiência aqui no pré-natal tem sido excelente. Tive complicações de saúde, mas, graças a Deus, os médicos me atenderam na hora certa*” (Camila, 23 anos, parda, grupo focal).

“*Medicamentos, não me faltaram. Eles me davam e, com isso, não tive problema*” (Fernanda, 19 anos, branca, grupo focal).

As mulheres multíparas que haviam passado pela experiência de assistência durante o pré-natal na Venezuela, antes da crise, apontaram diferenças entre os dois países. No Brasil, sentiram-se menos acolhidas e relataram ausência de exame físico. Outra queixa frequente foi relacionada ao modelo vigente de consultas intercaladas entre enfermeiros(as) e médicos(as).

“*Na Venezuela, a partir do 2º mês, começam a medir a barriga. Ontem fui fazer meu check-up, demorei mais esperando eles me atenderem e a única coisa que fizeram foi: ‘sua nova receita para você retirar sua vitamina’*” (Camila, 23 anos, parda, grupo focal).

“*Se eu quiser uma consulta com o médico, que é o que me interessa* (...) *primeiro me encaminham para a enfermeira. Muito legal, fazem os exames. Tenho que esperar a cada dois meses uma consulta com o médico, enquanto na Venezuela não era assim*” (Carolina, 29 anos, amarela, grupo focal).

### Acesso e assistência ao parto

Entre aquelas que vivenciaram um parto no Brasil nos 12 meses anteriores à pesquisa, 97,8% tiveram gestação única e realizaram o parto em maternidade. A maioria teve parto vaginal (57,9%), todas tiveram como desfecho nascidos vivos, sendo 14,6% com idade gestacional menor que 37 semanas. Em relação à assistência ao parto, 95,1% tiveram acompanhante de livre escolha durante a internação, sendo grande parte durante todo o tempo (91,4%). Mais de 14,1% das migrantes relataram alguma complicação durante o parto para as mães e 21,2% para os recém-nascidos (Tabela 3).

**Tabela 3:** Informações sobre o parto de mulheres migrantes venezuelanas residentes em Manaus, Amazonas, que tiveram filho no Brasil nos últimos 12 meses. ReGHID, 2021.

Informações	n	%	IC95%
Tipo de gestação			
Única	74	97,8	85,5-99,7
Gemelar	1	2,2	0,3-14,5
Tipo de parto			
Vaginal	44	57.9	45,1-69,8
Cesariana	31	42.1	30,2-54,9
Desfecho do parto			
Nascido vivo	75	100,0	100,0-100,0
Local do parto *			
Maternidade	73	98,9	92,3-99,8
Casa	1	1,1	0,2-7,7
Idade gestacional do parto (semanas)			
< 37	11	14,6	7,5-26,6
≥ 37	64	85,4	73,4-92,5
Teve acompanhante para o parto			
Sim	69	95,1	84,6-98,6
Não	6	4,9	1,4-15,4
Acompanhante ficou durante todo o tempo (n = 69)			
Sim	63	91,4	81,0-96,3
O acompanhante não quis ficar	1	0,7	0,1-5,3
A maternidade não deixou ficar	5	7,7	3,0-18,2
Complicação materna no parto			
Sim	11	14,1	7,5-25,1
Não	64	85,9	74,9-92,5
Complicação neonatal no parto			
Sim	16	21,2	12,5-33,8
Não	59	78,8	66,2-87,5

IC95%: intervalo de 95% de confiança.

* 1 respondeu a opção não sabe/não responde.

A respeito do acesso ao hospital para atendimento ao parto, as falas das entrevistadas revelaram histórias de peregrinação materna, relacionadas a fatores como a lotação, diferenças no nível de complexidade das maternidades e classificação de risco gestacional.

“*Me mandaram para a Maternidade B. Fui lá quando comecei a urinar sangue. Lá me disseram que não, que, se fosse uma emergência, não iam deixar; não tinha vaga, que eu tinha que ir para outra maternidade*” (Clara, 27 anos, não refere, grupo focal).

“*Tive que visitar todas as maternidades de Manaus, praticamente* (...) *fiquei uma semana com dor*” (Alma, 24 anos, branca, entrevista).

Apesar disso, após o ingresso nas maternidades, a maioria das participantes destacou a qualidade da atenção proporcionada pela equipe de saúde durante o trabalho de parto. As boas práticas - tais como medidas não farmacológicas para alívio da dor, contato pele a pele e amamentação na primeira hora de vida - foram ofertadas e bem recebidas. Poucas experiências negativas foram mencionadas e, quando isso ocorreu, geralmente estavam relacionadas às atitudes de membros específicos da equipe.

“*O atendimento lá foi excepcional. Eles me ajudaram com medidas para que a dor fosse menos intensa*” (Alma, 24 anos, branca, entrevista).

“*Quando tiraram a bebê, colocaram ela no meu peito*” (Valentina, 27 anos, negra, entrevista).

“*Por causa da própria médica, ela me tratou com má vontade, mas eu não prestei atenção nela*” (Nina, 28 anos, parda, entrevista).

As participantes relataram experiências positivas na hospitalização, destacando a disponibilidade de medicamentos, comida e cuidados ao recém-nascido. Elas também valorizaram os exames e a triagem neonatal como importantes para a assistência à criança.

“*Eles me deram quatro antibióticos. Uma boa alimentação. Estava tudo limpo, minha cama era confortável, tinha espaço onde colocar meu bebê, para ele ficar comigo. Excelente*” (Blanca, 35 anos, parda, entrevista).

“*A enfermeira o atendeu primeiro e encaminhou para o pediatra. Ela viu a cabecinha dele, olhou para ver se estava respirando bem, lhe pesaram e mediram* (...) *Fizeram-lhe o exame de ouvido, de vista, verificaram se ele estava fazendo cocô normalmente, examinaram a barriguinha, o peito, vacinaram, fizeram o teste do pezinho*” (Carla, 24 anos, parda, entrevista).

Diferenças no cuidado no momento do parto entre a Venezuela e o Brasil foram apontadas pelas entrevistadas. Como aspectos positivos, destacaram a possibilidade de realizar o parto sem a necessidade de custear os insumos e o fato de poderem estar acompanhadas, algo que referem não ser permitido na Venezuela.

“*Na Venezuela tinha que comprar tudo, minha medicação, antibióticos, remédios para dor, a sutura, tudo, comprei tudo*” (Blanca, 35 anos, parda, entrevista).

“*Na Venezuela não deixam você ter acompanhante, você fica sozinha. Nem sua mãe nem ninguém pode entrar. Aqui eu vi que foi bom, que aqui pelo menos eles podem entrar com você*” (Valentina, 27 anos, negra, entrevista).

Por outro lado, algumas práticas empregadas no Brasil, como o incentivo ao parto normal e a restrição do uso da episiotomia e da manobra de Kristeller, foram vistas como negativas por algumas das entrevistadas.

“*Eu dei à luz e não me cortaram nem nada, eu mesma tive que expulsar. Minha irmã, que estava comigo, me ajudou. Eu falei para ela ‘sobe aqui para eu conseguir’, porque eu sei, lá na Venezuela, como eles ajudam quando você não consegue dar à luz*” (Sofia, 24 anos, parda, grupo focal).

### Idioma como eixo transversal no atendimento de mulheres migrantes

A barreira linguística afetou as mulheres de forma abrangente em toda a sua trajetória assistencial, desde o acesso ao pré-natal até o cuidado ao parto, em diferentes momentos e dimensões do atendimento, afetando o acolhimento e o entendimento de orientações clínicas. Algumas mulheres manifestaram receio de prosseguir com o pré-natal, pois não conseguiam compreender o que os profissionais falavam. Isso levou, em alguns casos, apesar da carência financeira, à busca por serviços privados com médicos fluentes em espanhol.

“*Não fui mais porque eles falam seu idioma e eu não entendo nada*” (Anita, 26 anos, branca, grupo focal).

“*Ainda não aprendi muito português, então fizemos o sacrifício de pagar por algo particular para entender bem como é tudo com um* [médico] *colombiano*” (Eva, 31 anos, branca, entrevista).

Além disso, houve relatos de mulheres que se sentiram discriminadas por parte dos trabalhadores das unidades de saúde devido à diferença do idioma.

“*Me consultei com um médico que, antes de engravidar, eu fazia o controle com ele, e ele me tratava muito mal. Ele disse: ‘Você tem tempo aqui e tem que falar português perfeito, porque eu fui numa época dessas lá, para a Venezuela, e falo espanhol perfeito’*” (Jurema, 24 anos, parda, entrevista).

Em contrapartida, quando havia algum profissional fluente em espanhol, isso favorecia a continuidade do cuidado, e elas se sentiam mais confiantes para pedir maiores esclarecimentos e orientações.

“*O médico que estava de plantão, graças a Deus, falava três idiomas, inglês, espanhol e português, então, ele me perguntou qual idioma, então, eu falei ‘espanhol’, e ele me explicou a situação*” (Carla, 24 anos, parda, entrevista).

## Discussão

As trajetórias assistenciais das mulheres migrantes durante o pré-natal e o parto reforçam a importância do SUS no acesso à saúde. Os achados quantitativos e qualitativos deste estudo se complementam, permitindo uma compreensão aprofundada das experiências de mulheres venezuelanas. De forma geral, ambos indicam que o acesso à assistência durante o pré-natal e o parto foi garantido. Estudo realizado em Manaus corrobora esses resultados ao mostrar que venezuelanas têm acesso universal e gratuito ao SUS, inclusive para cuidados pré-natais, independentemente do status migratório ^13^.

Esse cenário difere de outros países da América Latina. Na Colômbia e no Peru - principais destinos de venezuelanos ^1^ - o acesso ao Sistema Geral de Seguridade Social em Saúde (SGSSS) e ao Sistema Integral de Saúde (SIS), respectivamente, exige *status* migratório regular e outros requisitos administrativos. Apesar de exceções legais para gestantes, não há garantia de continuidade do cuidado, e procedimentos ou hospitalizações podem ser cobrados ^16^. No presente estudo, todas as mulheres entrevistadas - mesmo em situação irregular - foram atendidas no pré-natal e no parto, evidenciando a importância de políticas globais que assegurem o acesso universal à saúde para migrantes.

O componente quantitativo revelou que 23% das mulheres migraram grávidas, iniciando sua trajetória assistencial na Venezuela, onde as dificuldades de atendimento eram grandes. Leal et al. ^4^ mostraram que a dificuldade no acesso à saúde foi o segundo motivo mais citado pelas mulheres para decisão de migrar da Venezuela para o Brasil.

Neste estudo, verificou-se que o pré-natal ocorreu majoritariamente nas UBS, sendo avaliado como satisfatório devido ao acolhimento e acesso gratuito a consultas e medicações. Em Roraima, mulheres venezuelanas também relataram experiências positivas, reforçando a importância do acesso universal e do atendimento humanizado ^5^. Leal et al. ^4^ constataram que o uso dos serviços de saúde pelas venezuelanas foi quase três vezes maior ao das brasileiras. Isso sugere que a estrutura mobilizada para atender o fluxo migratório na Região Norte, aliada à existência do SUS, facilitou o acesso à saúde dessa população.

Dados da pesquisa *Nascer no Brasil* ^17^, realizada com mulheres brasileiras, destacam que, em alguns lugares, o acesso ao pré-natal pelo SUS chega a 99,3%. No entanto, a qualidade da assistência ainda é insuficiente, principalmente na Região Norte, onde apenas 10,5% dos atendimentos de pré-natal foram considerados adequados. Há necessidade de melhorias em todos os indicadores preconizados pelo Ministério da Saúde para um pré-natal adequado, incluindo início precoce, número mínimo de consultas, exames essenciais e vinculação à maternidade, a fim de prevenir desfechos adversos para mãe e bebê ^17^.

No caso das migrantes venezuelanas que participaram deste estudo, embora a avaliação geral tenha sido positiva, o componente qualitativo revelou insatisfações relacionadas à assistência no pré-natal, especialmente o estranhamento quanto ao modelo de atendimento intercalado entre enfermeiros(as) e médicos(as). Também foram relatadas ausência de exame físico, falta de esclarecimento de dúvidas e dificuldade de acesso à ultrassonografia.

No Brasil, a porta de entrada para o pré-natal é a UBS, com consultas intercaladas entre enfermeiros(as) e médicos(as), conforme necessidades clínicas das gestantes ^14^. Esse modelo está em consonância com diretrizes do Ministério da Saúde ^14^ e da Organização Mundial da Saúde (OMS) ^18^, que reconhecem o papel estratégico das equipes multiprofissionais no cuidado continuado, humanizado e custo-efetivo durante o ciclo gravídico-puerperal. No entanto, o desconhecimento por parte das migrantes sobre o funcionamento do sistema de saúde no país anfitrião - aspecto amplamente documentado na literatura internacional ^19^ - representa um desafio adicional para o SUS, podendo gerar percepções negativas mesmo quando os atendimentos seguem protocolos nacionais e internacionais.

A qualidade do cuidado pré-natal perpassa diversos aspectos, incluindo a realização do exame físico da gestante, a comunicação eficaz e o vínculo profissional-usuária. Estudos com gestantes brasileiras apontaram fragilidades nessas dimensões ^20^. No caso das mulheres migrantes, a superação de barreiras linguísticas é indispensável para assegurar uma comunicação efetiva ^19^ ^,^ ^21^. Diante disso, torna-se ainda mais urgente garantir uma assistência integral que considere as especificidades das mulheres migrante, promova educação em saúde e fortaleça sua autonomia.

A maioria das participantes da pesquisa quantitativa relatou ter realizado ultrassonografia, enquanto no qualitativo, muitas mencionaram dificuldade de acesso. Isso demonstra a complementaridade da análise integrada, que permitiu compreender as experiências daquelas que não conseguiram realizar o exame. Pesquisas com migrantes venezuelanas em Boa Vista também apontaram dificuldades para realizar esse exame ^5^ ^,^ ^22^. A pesquisa *Nascer no Brasil* já indicava, na Região Norte, maior déficit na cobertura de ultrassonografia ^17^. É preciso destacar, no entanto, que somente a partir do final de junho de 2023 a ultrassonografia passou a ser ofertada para o pré-natal de risco habitual, o que pode explicar a insatisfação relatada com a falta de acesso ^23^.

A assistência ao parto das participantes deste estudo foi realizada majoritariamente em maternidades públicas, sendo 42,1% por cesariana. De acordo com os dados do SIH/SUS ^3^, a taxa de cesariana em Manaus foi de 42,8% no ano de 2021, o que reflete uma prática que excede a recomendação da OMS (ausência de benefício para a mãe e o bebê com proporções de cesarianas acima de 10% e 15%) ^24^. A elevada proporção de cesarianas na Região Norte, como observado em Manaus, é uma tendência nacional. Pires et al. ^25^ destacaram um aumento linear e contínuo nas cesarianas no Brasil, que, em 2018, já ocupava a segunda posição mundial, ocorrendo em 55,7% dos partos.

Boas práticas foram mencionadas pelas participantes nas entrevistas qualitativas, incluindo o auxílio no controle da dor no trabalho de parto, a oferta de alimentação, além do incentivo ao contato pele a pele e à amamentação. Esses achados estão em consonância com o estudo de Leal et al. ^26^, que identificou um aumento significativo no acesso a tecnologias apropriadas ao parto e nascimento, especialmente o uso de métodos não farmacológicos para alívio da dor e a oferta de alimentação durante o trabalho de parto nas regiões Norte e Nordeste.

Outra boa prática, garantida na maioria dos casos, conforme revelado tanto pelos dados quantitativos quanto qualitativos, foi o direito ao acompanhante durante o parto e a internação, reconhecido pelos benefícios de apoio emocional e físico à parturiente ^26^. Esse achado já havia sido demonstrado em outro estudo com migrantes venezuelanas em Roraima ^5^.

Entretanto, esse direito nem sempre foi respeitado. No componente quantitativo, cinco mulheres relatam que a presença do acompanhante foi negada pela maternidade, o que configura uma falha grave no reconhecimento dos direitos das mulheres. Já os dados qualitativos evidenciaram outra limitação no exercício desse direito, relacionada à ausência de rede de apoio no Brasil. Em alguns casos, o companheiro não pôde estar presente por cuidar dos outros filhos. Estudo com migrantes indianas na Austrália identificou experiências semelhantes, marcadas por sentimentos de solidão, angústia e culpa diante do afastamento de suas redes afetivas ^27^. Esses achados reforçam que a garantia do suporte social é crucial para uma vivência positiva do parto entre mulheres migrantes.

Algumas multíparas relataram insatisfação com práticas obstétricas no Brasil - como o incentivo ao parto vaginal, a restrição da episiotomia e a manobra de Kristeller -, por acreditarem que tais condutas poderiam reduzir a dor e acelerar o parto. Makuch et al. ^5^ também observaram a surpresa das migrantes venezuelanas diante do favorecimento ao parto vaginal em vez da cesariana, frequentemente esperada por elas. Essas percepções evidenciam a influência de experiências prévias, crenças individuais e do contexto sociocultural na avaliação da assistência obstétrica ^28^, reforçando a necessidade de escuta qualificada às trajetórias e crenças das gestantes.

No Brasil, o parto vaginal espontâneo tem sido valorizado com base em evidências que demonstram redução da morbimortalidade materna e neonatal, contribuindo para a diminuição de cesarianas desnecessárias ^17^ ^,^ ^24^ ^,^ ^25^. A episiotomia passou a ser indicada de forma seletiva, pois sua aplicação rotineira não oferece benefícios comprovados e pode causar complicações ^26^. Já a manobra de Kristeller é formalmente contraindicada por apresentar riscos maternos e fetais ^26^. A utilização dessas práticas, inclusive, pode configurar violência obstétrica ^29^.

A análise dos dados demonstrou que a vinculação à maternidade, embora orientada durante o pré-natal, não se concretizou para todas. Os dados quantitativos evidenciaram que quatro em cada cinco mulheres receberam orientações sobre a maternidade de referência durante o pré-natal na atenção primária à saúde, mas apenas metade conheceu a unidade antes do parto. O componente qualitativo ampliou essa compreensão, revelando que nos, relatos de peregrinação materna, as entrevistadas não haviam conhecido previamente a maternidade, mostrando que a orientação, por si só, não é suficiente sem ações efetivas de articulação entre os níveis de atenção.

Outro resultado do componente qualitativo que evidencia a dificuldade de comunicação entre os níveis de atenção foi que poucas mulheres haviam recebido orientações sobre os cuidados pós-alta, e nenhuma recebeu visitas domiciliares no pré-natal e/ou no puerpério.

A falta de orientação adequada e a recusa de atendimento resultaram em experiências de sofrimento para algumas mulheres, como Lia, que, apesar de ter realizado pré-natal no SUS, precisou recorrer ao setor privado para exames. Na última semana de gravidez e durante o trabalho de parto, peregrinou entre quatro maternidades sem ter suas necessidades acolhidas. Nas duas primeiras, apenas a ausência de dilatação foi avaliada, e ela foi orientada a voltar para casa; na terceira, recebeu medicação analgésica; naa quarta maternidade, foi admitida já com 8cm de dilatação e relatou ter um parto prolongado e difícil. O recém-nascido teve complicações respiratórias e necessidade de reanimação (Figura 2).

**Figura 2:** Trajetórias assistenciais no pré-natal e parto de mulheres migrantes venezuelanas: os casos de Lia e Eva. ReGHID, 2021-2023.

UBS: unidade básica de saúde; SUS: Sistema Único de Saúde. Fonte: elaboração própria, baseado em Medeiros et al. ^10^. Nota: representação das trajetórias de busca por atendimento de duas mulheres migrantes venezuelanas durante o pré-natal e o parto em uma cidade do norte do Brasil. O caso de Lia (à esquerda) mostra múltiplas buscas por cuidados tanto no setor público quanto no privado, incluindo quatro maternidades diferentes no momento do parto. O caso de Eva (à direita) evidencia a busca por pré-natal privado, em razão da barreira linguística, e a realização de exames pela rede pública, com dois atendimentos na mesma maternidade no momento do parto. As setas indicam o fluxo e os retornos aos serviços. Os números indicam a ordem cronológica das buscas por cuidado. As setas contínuas representam o fluxo de atendimento; as setas duplas indicam múltiplos retornos.

A peregrinação anteparto persiste no Brasil, especialmente na Região Norte, e está associada aos piores desfechos neonatais ^30^. Estudos apontam que essa experiência também afeta mulheres brasileiras ^17^ ^,^ ^30^, revelando fragilidades persistentes no SUS. Entre migrantes, a peregrinação pode decorrer da ausência de vinculação prévia à maternidade de referência, além barreiras financeiras, administrativas e culturais ^19^. A vinculação à maternidade durante o pré-natal é uma estratégia para garantir um cuidado integral, reduzir a peregrinação e oferecer segurança às gestantes ^17^.

A barreira do idioma também impacto a assistência, como no caso de Eva, que recorreu a consultas particulares no pré-natal, mas realizou alguns exames pelo SUS, pagando por aqueles que não estavam disponíveis. Durante o parto, recebeu atenção inicial de enfermeiras, mas, com a troca de turno, sentiu-se insegura e relatou falta de atenção para suas dores. Com plaquetas baixas, foi transferida para a emergência, onde sofreu episiotomia sem justificativa clara por parte da médica (Figura 2).

As barreiras linguísticas dificultam a troca de informações com os profissionais e o entendimento do funcionamento do sistema de saúde de países receptores ^19^ ^,^ ^21^. Esse cenário reforça a necessidade do fortalecimento do SUS em termos de financiamento, infraestrutura e capacitação para qualificação da atenção ao pré-natal e ao parto para esta população. Além disso, a discriminação linguística relatada pelas entrevistadas também foi observada em outros estudos, evidenciando a culpabilização dos pacientes pela não compreensão do português ^8^ ^,^ ^21^ ^,^ ^28^.

A discriminação pode atuar como um entrave significativo no acesso aos serviços de saúde, afastando o migrante dos seus direitos e impactando negativamente em sua vida. Estratégias para superar as barreiras linguísticas, como o auxílio de tradutores profissionais e/ou a formação e inclusão de migrantes como agentes comunitários de saúde, podem ajudar na relação entre pacientes e profissionais, aperfeiçoando o cuidado ofertado e a experiência com o sistema de saúde ^19^ ^,^ ^31^.

Essas trajetórias mostram como as dificuldades na comunicação entre profissionais de saúde e gestantes/parturientes, aliadas à falta de articulação entre os níveis de atenção, comprometem o cuidado integral. Para mulheres venezuelanas, que enfrentam desafios culturais e linguísticos, essas fragilidades do SUS tornam-se ainda mais complexas.

### Potencialidades e limitações do estudo

Entre as potencialidades do estudo, destacam-se a combinação das abordagens quantitativa e qualitativa, o uso do RDS na amostragem do componente quantitativo para alcançar uma população de difícil acesso e a reconstrução da trajetória assistencial em ambos os componentes, que possibilitou uma visão integrada do cuidado recebido. Como limitações, incluem-se a não inclusão de mulheres indígenas que não falavam português ou espanhol, o que pode ter restringido a compreensão de experiências específicas desse grupo, e a ausência de dados extraídos a partir da caderneta da gestante, considerando a possibilidade de viés de memória, principalmente entre aquelas cujo parto ocorreu há cerca de 12 meses.

## Conclusão

A partir deste estudo, foi possível identificar as características socioeconômicas, culturais e reprodutivas das mulheres venezuelanas migrantes no Brasil e conhecer as barreiras ao acesso à assistência à saúde. A análise direcionada ao ciclo gravídico-puerperal evidenciou a importância do SUS no acolhimento e na promoção à saúde da população migrante, oferecendo contribuições para a saúde pública ao identificar suas necessidades e propor direcionamentos para o aprimoraramento da assistência.

A trajetória assistencial foi marcada por pontos positivos e negativos. As entrevistadas se mostraram, de maneira geral, satisfeitas com os cuidados durante o pré-natal e o parto, com acesso a consultas, exames, medicamentos e a boas práticas, como a presença do acompanhante, contato pele a pele e o incentivo ao aleitamento materno. No entanto, apesar dessa boa avaliação, também foram relatados problemas já existentes no SUS, como dificuldades na vinculação à maternidade durante o pré-natal, a peregrinação materna e falhas na integração entre os níveis de atenção. Somado a isso, a barreira linguística atuou como eixo transversal, impactando todas as fases do cuidado e intensificando os desafios.

## Data Availability

Os dados de pesquisa estão disponíveis mediante solicitação à autora de correspondência.
